# Supplementary material for: MetaRibo-Seq measures translation in microbiomes
Source: Nat Commun. 2020 Jun 29;11:3268. doi: 10.1038/s41467-020-17081-z (PMC7324362; doi:10.1038/s41467-020-17081-z)
Supplement: Supplementary file 10 — Supplementary Data 7 [file 41467_2020_17081_MOESM10_ESM.zip › File2/Confidence_High_Taxonomy/179766_out.krona.html]

Javascript must be enabled to view this page.

members
magnitude
magnitudeUnassigned
count
unassigned
taxon
rank

179766\_out

1

1
2759
superkingdom

1
kingdom
4751

1
451864
subkingdom

1
5204
phylum

29000
1
subphylum

162480
1
class

order
1
48846

1
54740
family

1
genus
643909


SRS013687\_contig\_number\_14985
species
1
5440
